# The effectiveness of nicotine replacement therapy on oral smokeless tobacco cessation and reduction rate: A systematic review

**DOI:** 10.18332/tpc/208023

**Published:** 2025-07-31

**Authors:** Ibtisam Moafa

**Affiliations:** 1 Department of Preventive Dental Sciences, Jazan University, Jazan, Saudi Arabia

**Keywords:** community health, smokeless-tobacco, cessation, oral cancer, nicotine replacement therapy

## Abstract

**INTRODUCTION:**

Oral smokeless tobacco (OST) is a major preventable risk factor for oral cancer. Nicotine replacement therapy (NRT), a commonly used pharmacological treatment in tobacco cessation interventions that help in reducing the withdrawal symptoms that individuals might experience in their attempt to quit. This systematic review aimed to assess NRT's effectiveness on OST cessation and reduction rates, addressing gaps in prior studies by incorporating recent research across diverse populations, including developing and developed countries.

**METHODS:**

A systematic search was conducted across PubMed, Cochrane, Embase, and Web of Science. The inclusion criteria were: English articles (2004 to June 2024), OST users of both genders, NRT intervention, control group (generic, placebo, or no intervention), and a 7-day point prevalence of OST abstinence and reduction at week 12. Studies were excluded if they lacked relevant outcomes, were non-English, or published before 2004. Data abstraction forms were used to extract study characteristics and results. The Practical Meta-Analysis Effect Size Calculator determined the effect sizes and directions. The risk of bias was assessed using Cochrane Collaboration’s tool.

**RESULTS:**

Eleven studies were included with 40–1067 sample size. Seven were effective on OST abstinence, with effect sizes ranging from d=0.10 to d=0.54 and odds ratios from OR=1.67 to OR=4.10. One study demonstrated OST reduction (d=0.16 for dips/day and d=0.17 for cans/week). Nicotine dependence, NRT dosage, self-efficacy and social support were identified as key factors influencing the NRT’s effectiveness in the included studies.

**CONCLUSIONS:**

NRT can aid in OST cessation. Combination of NRT with other interventions such as coach calls and web-based interactive setting can enhance the OST abstinence rate. The evidence, though promising, is limited by study variability, and inconsistent outcome reporting. Future studies should explore self-efficacy, social support, and NRT dosage with larger sample sizes for better assessment.

## INTRODUCTION

Oral cancer (OC) is the 6th most common cancer worldwide^1^. High incidence rates of OC were reported from developing countries in Asia, from countries in the middle and the south of the African continent, and from developed countries such as Australia, Russia, European countries, and countries in American continents^2,3^. The Arabian Peninsula countries, such as Saudi Arabia and Yemen, had reported high incident rates of OC as well^4^.

In the above-mentioned regions, oral smokeless tobacco (OST), which is the most preventable risk factor that is highly associated with OC, is readily available and overtly utilized by the inhabitants^5^. Indeed, data from these countries showed a raised risk of oral cancer because of OST use^6^. OST is a type of tobacco that is not burned or smoked, but one that is usually consumed by putting the product against the mucosal sites in the oral or nasal cavities, from which the nicotine can be absorbed into the body^5^. OST is available in different forms and products, e.g. snus, toombak, betel quid, and shammah^5,7^. OST has been promoted as a way for cigarette smokers to reduce the harms associated with smoking^8^. Studies have shown that more than 25 cancer-causing compounds are identified in OST^5,9^.

For the motive of tackling this dreadful disease (oral cancer), there is an increase in the national and international considerations towards OC risk factors prevention and intervention programs, which are typically developed and implemented based on the variations of the pervasive OST in each region. Nicotine replacement therapy (NRT) is one of the options that have been used for smoking and smokeless tobacco cessation. The aim of NRT is to replace the nicotine from the smokeless tobacco to reduce withdrawal symptoms that individuals might experience in their attempt to stop smokeless tobacco use, thereby helping in the process towards abstinence^10^. NRTs are available in different forms, such as nicotine gum and nicotine patches (16- or 24-hour release). Several studies have been conducted to test the effectiveness of NRTs, with mixed results^11,12^. A previous systematic review found that nicotine patches or gum did not receive much evidence to help individuals abstain from using smokeless tobacco; however, for nicotine lozenge, some evidence shows that it might be helpful^10,11^. Despite the rising in OST cessation interventions that are implemented on different populations around the world, only two systematic reviews have been conducted to assess the effectiveness of these programs^10,12^. However, one of these was limited to only the population of south Asia, whereas the other one assessed interventions carried out only in the USA and Sweden. The aim of this systematic review was to assess the effectiveness of NRT on the reduction and quitting rate of ST. For this reason, the research question of this systematic review was: ‘What is the effectiveness of NRT on the reduction and quitting rates of OST?’. This review attempted to fill the gap of abovementioned systematic reviews with more recent research and by assessing a broader population, developing and developed countries.

## METHODS

### Literature search

A systematic search was conducted across PubMed (1–4 April 2024), Cochrane Library (5–8 April 2024), Embase (1–5 May 2024), and Web of Science (6–10 May 2024), using MeSH terms and free-text keywords related to nicotine replacement therapy, smokeless tobacco, cessation, and reduction. Supplementary searches were conducted via citation tracking and Google Scholar (12–14 May 2024). A final update search (27–30 May 2024) was carried out to ensure the inclusion of the most recent evidence.

### Eligibility criteria

The inclusion criteria have been formulated according to the components of PICOS (Population, Intervention, Comparison, Outcomes and Study Design). Studies included in the review had to satisfy the inclusion criteria, shown in Supplementary file Table 1. Studies were included if they involved: OST users of any gender (population), assessed NRT as the intervention, had placebo, no intervention, or other behavioral support interventions (comparison), reported 7-day point prevalence OST abstinence or reduction at week 12 (outcome) and published randomized and non-randomized trials in English between 2004 and June 2024 (study designs). Studies were excluded if they lacked relevant outcomes, were non-English, or published before 2004. For synthesis, studies were grouped based on whether they reported OST cessation, reduction, or both, and further categorized by intervention type and setting.

### Selection process

The study selection process followed the PRISMA flow diagram. Duplicates were removed using EndNote prior to screening. Two reviewers independently assessed the remaining articles through a two-phase screening process: first by evaluating titles and abstracts, followed by full-text review based on the predefined eligibility criteria. Disagreements between reviewers were resolved through discussion, with input from a third reviewer when necessary, ensuring a consistent and unbiased selection process.

### Data collection process

A data abstraction form was used for extracting the data from the studies that met all the inclusion criteria and to systematically collect the relevant information from the studies by two independent reviewers. The form was constructed from the data abstraction form developed by Zaza et al.^13^. The relevant points from the Zaza et al.^13^ form were retrieved and used in developing the abstraction form. The form consisted of three tables to collect the information of the characteristics, results, and risk of bias. The characteristics table included information about the author, country, year, publication state, study design, participants’ characteristics, description for the interventions, description for the comparison group, outcome measure, measuring instrument, time point and space for notes. While the second table involved analysis, covariates, information about outcomes for the intervention and control group, effect size and direction of effect. The third table was about the risk of bias and consisted of information regarding the selection bias, performance bias, detection bias, attrition bias, reporting bias and whole articles summary judgment. The data abstraction form can be found in the Supplementary file Material 1.

### Quality assessment

Cochrane Collaboration’s tool for assessing the risk of bias was utilized to determine the risk of bias for each study^14^. The tool illustrates a recommended approach for assessing the risk of bias in the domains: selection bias, performance bias, detection bias, attrition bias and reporting bias. It also provides criteria to judge the risk of bias by selecting a judgment of ‘unclear risk’, ‘high risk’ and ‘low risk’ of bias per individual domain^14^. Supplementary file Table 2 illustrated the summary of judgments as determined by the risk of bias number for each study. The low-risk column set as a reference where the amount of high risk and unclear risk will be relating to it. The number of risks of bias ranged from 0 to 5 according to the aforementioned five risk of bias domains demonstrated in Cochrane Collaboration. Three was the cutoff point which determines the summary of judgment. In other words, if the amount of low risk of bias was 4 or 5 (>3), then the summary judgment will be low risk. The same measurement was obtained for the unclear and high risk of bias summary judgment. While if the amount of low risk of bias was ≤3, then the summary judgment will be based on the number of other types of risk of bias which could lead to intermediate risk, high risk and unclear risk.

### Data analysis

Different ways were used to analyze the results of the studies. The intervention outcome was presented as rates regarding OST abstinence and reduction. Cohen’s d and odds ratio (OR) were used for the effect size interpretation. The effect value interpreted as large, medium and small as shown in Supplementary file Table 3. To compare the likelihood of participants in the intervention group who did quit or reduced the OST consumption in contrast to those in the non-intervention group, an odds ratio value was described^15^. Effect size calculation was done using web-based effect size calculator (Practical Meta-Analysis Effect Size Calculator)^16^.

## RESULTS

### Study selection

**Figure 1:**
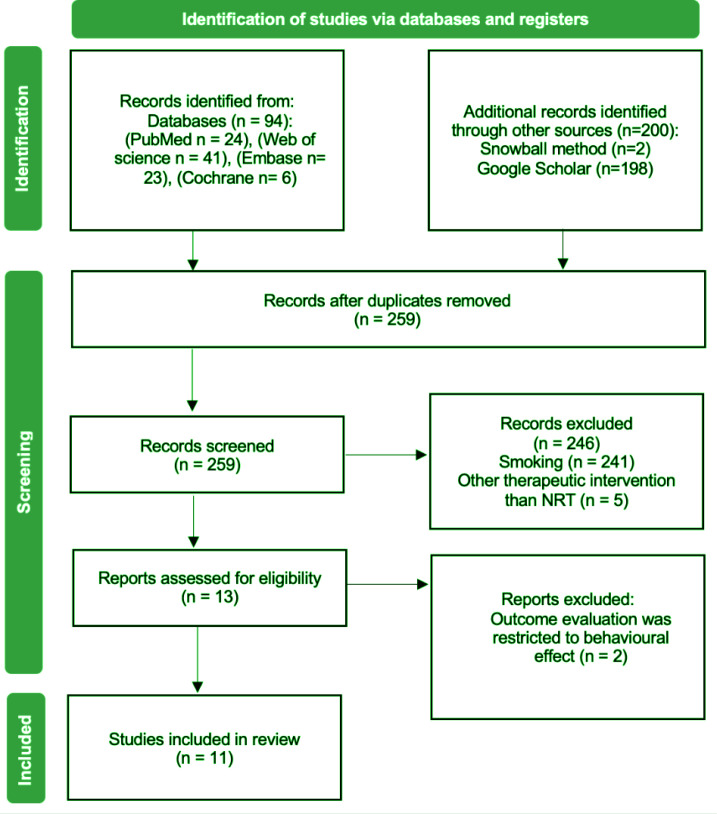
PRISMA flow diagram for literature selection process

As described in Methods section, the database searching generated 94 articles. The additional studies identified through snowball sampling were two studies. Studies identified using Google Scholar were 198. After removing the duplicates, the number of retrieved studies were 259. Following that, title and abstract screening were done to end by excluding 246 articles, in which 241 studies were measuring smoking tobacco as an outcome only without assessing OST abstinence. There were five studies that used OST cessation therapies other than NRT, such as varenicline and bupropion as illustrated in Figure 1. After that, the remaining 13 articles were subjected to full-text assessment, resulting in the exclusion of two articles which focused on the behavioral part of the intervention with no data regarding the effect of NRT. Finally, the remaining eleven articles were included in the review.

### Study characteristics

The study characteristics are shown Table 1. The review included eleven published studies in the USA, India, Pakistan, Bangladesh and Sweden^17-27^, all were randomized controlled trials, except one study which was a prospective nonrandomized trial^27^. Different settings were identified in the included studies such as home, work, health care and web-based setting. There were no differences in the sample size and dropout rates between the experimental group and the control group as reported by the articles’ authors and re-evaluated by the review author using the CONSORT diagram and the full-text assessment of the included studies. The dropout rate of the studies was between 20% and 34%. The participants’ sample size ranged from 40 to 1067. Many of the included studies had male participants only, and when female participants were present, they were in the placebo group only. Most of the participants were white Caucasian in the USA studies. Different OST products such as kahani, pan masala, Grizzly, Copenhagen, Skoal, and Kodiak were commonly utilized by the study participants. Nicotine patch and nicotine lozenge were the used materials in NRT intervention. While for the comparison; four studies included placebo^18,20,21,26^, two studies included oral health education^22,23^, one study included coach calls^24^, one study used tobacco-free snuff^25^, one study used web-based interactive^17^, one study compared different doses of NRT^21^ and one study with no comparison group^27^. Ten studies measured OST abstinence rate outcome and only two studies measured OST reduction rate^17,25^. While the instrument used for outcome measurement was varied from depending on self-report and dairies either written or electronic solely or accompanied with validation test such as taking urine specimen for anabasine and antabine as a biochemical confirmation of OST abstinence, Fagerström test for nicotine dependence-OST (FTND-ST) which was modified to specifically measure OST nicotine dependence level, or the OST abstinence rate was validated with saliva cotinine test.

**Table 1:** Study design, participant characteristics, intervention components and outcome measures of the included trials

*Authors Year Country*	*Design*	*Participants*	*Interventions*	*Outcomes*	*Instrument*	*Time point*	*Notes*
Ebbert et al.^21^ 2007 USA	RCT	42 male ST users consume at least 3 cans or pouches per week	Intervention: Three intervention groups with different high dose of nicotine patch: 63, 42 or 21 Placebo	7day point prevalence ST abstinence at week 12	Self-report, urine specimen for anabasine as biochemical confirmation of tobacco abstinence	At 8 weeks, 12 weeks and 6 months	
Rajja et al.^23^ 2016 India	RCT	40 male participants with history of ST consumption within the past 30 days	Intervention: Nicotine replacement therapy (NRT) group Oral Health Education (OHE) group	ST abstinence at week 12	Self-report, Nano-CheckTM Rapid Nicotine test was used for the qualitative detection of cotinine in urine.	Follow up at intervals of 1 week, 2 weeks, 1month, 2 months and 3 months	
Ebbert et al.^26^ 2010 USA	RCT	60 male participants reported ST as their primary tobacco of use and had used ST daily for at least 6 months and wanted to quit	Intervention: Mailed 4 mg nicotine lozenge and self-help assistance Placebo including only self-help assistance	7-days point prevalence ST abstinence at week 12	Self-reported, daily record of withdrawal symptom and medications using modified Minnesota Nicotine Withdrawal Scale MNWS	At baseline, at 12 weeks, at 6 months	
Schiller et al.^19^ 2012 USA	RCT	199 male ST users aged ≥18 years, interested in reducing ST use but not quitting and having an established date for quitting within the next 90 days	Immediate ST cessation group: set a quit date soon after enrollment and were offered nicotine patch therapy to help in their cessation efforts (n=97) ST reduction group: provided with their choice of either 4 mg nicotine lozenge or ST brand switching to help them reduce their ST use or levels of nicotine exposure. n=102	7-day point prevalence ST abstinence at week 12	Self-report, phone call, tobacco use diaries, urinary anatabine was used to verify abstinence in subjects receiving NRT	12 weeks, 26 weeks and at 26 weeks post enrollment	
Ebbert et al.^25^ 2013b USA	RCT	52 ST users aged 18–55 years, daily ST use for the past 12 months; identified ST as their primary tobacco product; used an average of ≥3 cans/pouches per week and with general good health	Intervention: Daily dose of 42 mg of nicotine patch (n=25) Placebo patches (n=27)	7-day point prevalence ST abstinence at week 12	Self-reported, urinary anabasine concentration of <2 ng/mL, Fagerström test for nicotine dependence-ST (FTND-ST)	At baseline, at 3 months and at 6 months	Dropout 34%
Severson et al.^24^ 2015 USA	RCT	1067 ST users aged ≥18 years with daily use of ST for at least 1 year and willing to quit in the next month	Nicotine lozenge group Coach calls group Nicotine lozenge and coach calls group	7-days point prevalence smokeless tobacco abstinence at week 12	Self-report	At 3 and 6 months	Dropout 20%
Ebbert et al.^18^ 2013a USA	RCT	81 ST users aged ≥18 years with daily use of ST for at least 1 year and had no plan to quit in the next month.	Nicotine lozenge group (n=40) Tobacco-free snuff group (n=41)	Percentage of ST reduction at week 12	Self-report, (FTND-ST), urinary anabasine and anatabine concentrations of <2 ng/mL	Follow up at intervals of 1 week, 2 weeks, 1month, 2 months and 3 months	Dropout 28%
Danaher et al.^17^ 2015 USA	RCT	407 ST users, aged ≥18 years, use ST daily for at least 1 year, agreed to quit using tobacco within the next month, US resident	Intervention: Interactive MyLastDip web-based intervention + nicotine lozenge group (n=205) Interactive MyLastDip web-based intervention only group (n=202)	7-day point prevalence ST abstinence at week 12 Reduced ST use (number of cans/pouch used per week) at week 12	Self-report	Assessment at 3 months and 6 months after enrollment	
Ebbert et al.^20^ 2009 USA	RCT	270 participants, aged ≥18 years, ST as their primary tobacco, wanted to quit, had used ST daily for at least 6 months, and were in good general health	Intervention: active lozenge (n=136) Placebo (n=134)	7day point prevalence ST abstinence rate at week 12	Self-report, urinary anabasine for biochemical validation of self-reported abstinence	At 3 and 6 months	
Siddiqui et al.^22^ 2024 Bangladesh, India and Pakistan	RCT	264 participants aged >18 years daily ST users willing to make a quit attempt within 30 days; 88 from Pakistan, 44 form India and 132 from Bangladesh	Intervention A: 4 or 6 mg NRT chewing gum for 8 weeks (n=66) Intervention B: BISCA, face-to-face behavioral support for South Asian ST cessation (n=66) Combination of Interventions A and B (n=66) 4. Control condition: very brief advice to quit (VBA) (n=66)	7-day point prevalence ST abstinence rate at week 12	Self-report	Follow up at 6, 12 and 26 weeks	
Walstrom et al.^27^ 2010 Sweden	Prospective Non-randomized trial	50 participants, daily snus use of >2 cans/week (>100 g snus) for >10 years	A short motivational information-giving session with the unambiguous message that snus is harmful to health, a supply of NRT	Abstinence from any tobacco use from the second visit until the endpoint at 12 months	For biochemical verification of self-reported tobacco use, saliva cotinine was used	Follow up at 2 weeks, 6 weeks, 3 months, 6 months and 12 months	No control group

### Quality assessment

Six studies were identified with low risk of bias^17-22^. Three studies were identified with an unclear risk of bias^23-25^. While there was one study with an intermediate risk^26^, and only one study with a high risk of bias^27^. The study with high risk of bias included high risk in selection bias, in detection bias and in reporting bias. A detailed quality assessment information for the studies can be found in Table 2.

**Table 2:** Quality and risk of bias assessment of included studies based on methodological criteria

*Authors Year Country*	*Selection bias*	*Performance bias*	*Detection bias*	*Attrition bias*	*Reporting bias*	*Summary risk of bias*
Raja et al.^23^ 2016 India	Low risk Quote: ‘The patients were randomly assigned using lottery system’	Unclear risk	Unclear risk	Unclear risk	Low risk	2LR and 3UR = Unclear risk
Danaher et al.^17^ 2015 USA	Low risk	Low risk	Low risk	Low risk	High risk Quote: ‘The results were similar for smokeless tobacco abstinence’	4LR and 1HR = Low risk
Severson et al.^24^ 2015	Unclear risk Quote: ‘Participants were randomly assigned to one of three conditions’	Unclear risk	Unclear risk	Low risk: Quote: ‘Because we recruited many participants from across the country our study may be considered an effectiveness trial’	Low risk	2LR and 3UR = Unclear risk
Ebbert et al.^25^ 2013b USA	Low risk Quote: ‘Subjects were randomly assigned in a 1:1 ratio to the treatment condition using a computer-generated randomization sequence with a block size of two’	Low risk Quote: ‘All study medication was labeled and dispensed according to subject identification ensuring that treatment assignment remained concealed to the subject, investigator, and all study personnel’	Low risk	Unclear risk	Low risk	4LR and 1UR = Low risk
Ebbert et al.^18^ 2013a USA	Unclear risk Quote: ‘Our study was a randomized, two-group, multicenter pilot clinical trial with a 12-week medication phase and follow-up through 6 months after randomization’	Unclear risk	Unclear risk	Low risk	Low risk	2LR and 3UR = Unclear risk
Schiller et al.^19^ 2012 USA	Low risk	Low risk	Low risk	Low risk Quote: ‘We found no difference in dropout rate between the immediate cessation group and reduction group’	High risk The authors did not report mean, SD or CI at 3 months	4LR and 1HR = Low risk
Ebbert et al.^26^ 2010 USA	Unclear risk Quote: ‘Subjects were randomized to the 4 mg nicotine lozenge or placebo for 12 weeks with follow-up to 6 months’	Low risk Quote: ‘Although study assistants were blinded to treatment assignment and all subjects received ASH, abstinence rates may be inflated but likely not differentially by group’	High risk Quote: ‘Study assistants providing counseling also performed outcome assessments’	Unclear risk	Low risk	2LR, 2UR and 1HR = Intermediate risk
Ebbert et al.^20^ 2009 USA	Low risk Quote: ‘A computer-generated randomization sequence assigned participants in a 1:1 ratio to treatment condition with a block size of four stratified by site’	Low risk Quote: ‘Study participants, investigators, and all other study staff were blinded to treatment assignment’	Low risk	Low risk	Low risk	5 LR = Low risk
Ebbert et al.^21^ 2007 USA	Low risk Quotes: ‘Study personnel who did not have subject contact used the randomization schedule to dispense the appropriate study patches into containers labeled according to subject identification number’ ‘Patches were distributed from a central pharmacy’	Low risk Quotes: ‘Group assignment with allocation concealment was determined by a randomization schedule, and subjects were assigned the next sequential subject identification number upon arrival’	Low risk	Low risk None of the participants dropped out of the study	Low risk	5 LR = Low risk
Siddiqui et al.^22^ 2024 Bangladesh, India and Pakistan	Low risk Quote: ‘Participants were randomized to one of four trial arms in an equal allocation ratio using a country-stratified, permuted block randomization with varying block sizes using R software by the trial statistician and shared with the respective trial coordinators, who assigned participants to the interventions. Participants within each country and within each study site were randomized independently into one of the four arms of the study’	Low risk Quote: ‘Opaque, sealed envelopes were used to conceal treatment allocation up to the point of randomization’	High risk Quote: ‘Blinding of participants and research staff carrying out followup assessments was not carried out due to the pilot nature of the study and resource limitations at each site; however, the statistician was blinded until after the main analysis was conducted’	Low risk Retention rates were 94.7% at 6 weeks	Low risk	4 LR and 1 HR = Low risk
Wallström et al.^27^ 2010 Sweden	High risk Quote: ‘The study was designed as a prospective, open-label, non-randomized, interventional trial, the study lacked control groups and had a relatively small sample size, selection of highly motivated subjects’	Unclear risk Quote: ‘All subjects were informed by telephone about the cessation program and then gave their oral consent to participation’	High risk Quote: ‘All subjects were examined and reviewed by the same investigator’	Low risk Quote: ‘Forty-five of the 50 subjects who entered the study completed the program	High risk The authors did not report data of the mean, SD, CI or p value at 3 months	1LR, 1UR and 3HR = High risk

### Results across studies

Among the eleven studies included in this review, only two studies – Ebbert et al.^18^ and Raja et al.^23^ – reported a moderate positive effect of NRT on OST abstinence at week 12, with effect sizes of OR=4.10 and Cohen’s d=0.54, respectively^18,23^. Ebbert et al.^18^ conducted their study in the United States, using high-dose nicotine patches, while Raja et al.^23^ conducted their study in India, examining standard NRT gum or lozenges. In contrast, five studies – Danaher et al.^17^, Ebbert et al.^20^, Siddiqui et al.^22^, Severson et al.^24^, and Ebbert et al.^25^ –reported a small but positive effect of NRT on OST abstinence or reduction rates, with odds ratios ranging from 1.67 to 2.32 and effect sizes from d=0.10 to 0.17. Of these, four studies showed statistically significant differences between the intervention and control groups^17,20,22,24^, while one did not reach statistical significance. Two studies – Schiller et al.^19^ and Wallström et al.^27^ – did not report outcomes of OST abstinence at week 12. However, Schiller et al.^19^ mentioned a significant difference in cessation between groups, though without adequate data for effect size calculation. Additionally, two other studies – Ebbert et al.^26^ and Ebbert et al.^21^ – provided insufficient data to calculate effect sizes.

Across the included studies, a range of covariates were considered, including age, gender, ethnicity, education, self-efficacy, peer support, alcohol and tobacco use, nicotine dependence, NRT dosage, depression, BMI, and physiological measures such as blood pressure and cholesterol. Notably, self-efficacy, social support, NRT dosage, and high nicotine dependence were consistently associated with greater NRT effectiveness in promoting OST abstinence. Statistical methods used to analyze outcomes included t-tests, ANOVA, chi-squared tests, Fisher’s exact test, and various regression models. A complete summary of individual study outcomes is presented in Table 3.

**Table 3:** Summary of outcomes, statistical results, effect sizes, and direction of effects for NRT interventions across included studies

*Authors Year Country*	*Analysis*	*Co-variates used*	*Results for outcome*	*Effect size*	*Direction of effect*
Raja et al.^23^ 2016 India	Unpaired t-test was used for intergroup comparisons	Not mentioned	At week 12: ST abstinence; intervention (NRT*) Mean=1.65 comparison (OHI) Mean=2.25 p>0.05	‘Medium’ Cohen’s d	There was a positive effect of NRT on ST abstinence
Danaher et al.^17^ 2015 USA	ST abstinence was assessed including analysis of complete cases and intent-to-treat (ITT) imputation analysis	Age, gender, marital status, race and ethnicity, education, selfefficacy, expected support from the partner, depression status	At week 12: ST abstinence; 39.8% (160/407) 95% CI: 1.206–2.734 p=0.004	‘Small’ OR=1.81	There was a positive effect of web based combined with NRT on ST abstinence
	ST reduction was assessed using ANOVA and chi-squared analyses		At week 12: ST reduction; Cans/week 76% Authors mentioned that the rate did not differ by condition	Authors did not report the required data for the effect size calculation	
Ebbert et al.^25^ 2013b USA	Logistic regression for tobacco abstinence at 3 months 90% confidence interval for the odds ratio	Age, gender, ethnicity, marital status, level of education, confidence in not using ST, alcohol drinks and binge drinking episodes	At week 12: ST abstinence; Intervention group: 12 (48%), Placebo: 5 (19%) 95% CI: 1.4–11.46 p=0.014	‘Medium’ OR=4.10	There was a positive effect of nicotine patch on ST abstinence
Ebbert et al.^18^ 2013a USA	For all treatment comparisons of tobacco reduction and abstinence outcomes, the Fisher exact test with p<0.20 was used and for the percentage change from baseline is compared between groups using the rank sum test	Not mentioned	At week 12: ST abstinence; nicotine lozenge 20% tobacco-free snuff 12% one-tailed p=0.257	‘Small’ Cohen’s d=0.105	There was a positive effect of NRT* on ST abstinence
			At week 12: ST reduction ≥50% Dips/day nicotine lozenge 59% tobacco-free snuff 66% 95% CI : -0.273–0.6053 p=0.809	‘Small’ Cohen’s d=0.166	There was a positive effect of NRT on reduction of Dips/day use of ST
			At week 12: ST reduction ≥50% Cans/week nicotine lozenge 55% tobacco-free snuff 66% 95% CI: -0.257–0.615 p=0.890	‘Small’ Cohen’s d=0.178	There was a positive effect of NRT on reduction of Cans/week use of ST
Severson et al.^24^ 2014 USA	ANOVA and chi-squared test were used to evaluate baseline equivalence across the three arms of the study Regression models with covariate adjustment for baseline tobacco use were used to examine reduced ST usage among participants	Age, gender, ethnicity, marital status, level of education, confidence in not using ST, readiness, expected support from other, best friends using ST	At week 12: ST abstinence; Nicotine lozenge + coach call versus coach call 95% CI: 1.56–2.84 p<0.001	‘Small’ OR=2.11	There was a positive effect of nicotine lozenge combined with coach calls on ST abstinence
			At week 12: ST abstinence; Nicotine lozenge + coach call versus lozenge alone 95% CI: 1.24–2.24 p<0.001	‘Small’ OR=1.67	There was a positive effect of nicotine lozenge alone on ST abstinence
			At week 12: ST abstinence; Nicotine lozenge versus coach call OR=1.26; 95% CI: 0.93–1.70 p=0.415		
Schiller et al.^19^ 2012 USA	To compare different treatment groups, t-test and Wilcoxon rank-sum test were used. Linear mixed models were used for analyzing the amount of ST use at different time points adjusting for the background level	Not mentioned	At week 12: ST abstinence; Was higher among those in the immediate cessation versus reduction group (p=0.04)	Authors did not report the required data for the effect size calculation	
			At week 12: ST abstinence; Significant reductions among non-quitters were observed for both groups (p<0.0001) with no differences between groups	Authors did not report the required data for the effect size calculation	
Ebbert et al.^26^ 2010 USA	Lozenge use was compared between groups using the rank sum test. Tobacco abstinence endpoints were summarized and compared using the chisquare test	Not mentioned	At week 12: ST abstinence; 47% in the nicotine lozenge 37% in placebo OR=1.509 p=0.432	Authors did not report the required data for the effect size calculation	
Ebbert et al.^20^ 2009 USA	Tobacco abstinence endpoint was analyzed using logistic regression	Not mentioned	At week 12: ST abstinence; 36% lozenge 27.6% placebo 95% CI : 1.2–3.2 p=0.013	‘Small’ OR=2.0	There was a positive effect of nicotine lozenge on ST abstinence
Ebbert et al.^21^ 2007 USA	Analyses were performed using logistic regression with tobacco abstinence as the dependent variable and nicotine patch dose as the independent variable	Not mentioned	At week12: ST abstinence; *IG a =21 mg: 40%, IG b =42 mg: 73%, IG c =63 mg: 70% Placebo 73% p=0.74, OR=1.1 No confidence interval reported *IG=Intervention group	Authors did not report the required data for the effect size calculation	
Siddiqui et al.^22^ 2024 Bangladesh, India and Pakistan	Log-binomial regression was used to estimate treatment effects	Not mentioned	At week 12: ST abstinence; NRT* 65.2% Very brief advice 37.9% BISCA 57.6% BISCA + NRT 62.1% (84/132) OR=2.32; 95% CI: 1.31–4.1	‘Small’ OR=2.32	Indicated preliminary effects for NRT (compared to no NRT)
Wallström et al.^27^ 2010 Sweden	The Mann–Whitney U-test was used to detect differences for non-paired data The Wilcoxon signed-rank test was used for paired data For correlation analyses, linear regression was used	BMI, blood pressure, cholesterol	At week 12: ST abstinence; 58% reported quitting 42% failure to quit	Authors did not report the required data for the effect size calculation	

* NRT: nicotine replacement therapy.

## DISCUSSION

This systematic review assessed the effectiveness of NRT intervention on OST cessation and reduction rate. With the aim that if NRT is effective, it will aid in prevention and reduction of OC incidence rate. The review included eleven articles published between 2004 and 2024. Of all the articles included in this review, seven interventions were identified as effective on OST cessation. In these interventions, the effect was positive and ranged from small to medium. The effect was associated with statistically significant difference between the experimental group and the control group in most of those interventions. Only one intervention reported the effect of NRT on OST reduction which was positive and small with no statistically significant difference between the experimental group and the control group.

When comparing the effect sizes to the risk of bias assigned in the quality assessment, most of the interventions with low and moderate effect size both have been qualified as interventions with low risk of bias. Unclear risk of bias was also qualified for interventions with low and moderate effect sizes. It can, therefore, be said that there was no apparent relationship between the effect size and the quality of the studies. Moderate effect sizes were found in studies with small sample size and were in healthcare and workplace settings. The interventions with a larger sample size were associated with a smaller effect size and were conducted at home and combined with behavioral interventions such as coach calls and web-based interactive sessions. This indicates that both sample size and the context in which the intervention was delivered can influence the effect size^28^.

Different aspects could explain the efficacy of NRT on OST cessation found in the included interventions. It could rely to some extent on OST users being dependent on nicotine. When participants had a high nicotine dependence level at baseline, NRT was associated with more OST abstinence rate in comparison to the other participants with moderate or low nicotine dependence level. This could explain the moderate effect of NRT which was illustrated in one intervention^23^. In comparison to the control group, NRT group was associated with higher dependence level at baseline; lower dependence level and better performance in OST quitting at week 12. A similar finding was shown in the aforementioned systematic review^29^.

Other aspects which could contribute to the effectiveness of NRT were self-efficacy and social support^30-32^. NRT was associated with increased OST abstinence rate among the participants who had a high confidence in their ability to quit and provided with support either through an interactive web-based intervention or coach calls. NRT was associated with low OST abstinence rate when participants had a low self-efficacy. This was the case in a study done by Danaher et al.^17^. Self-efficacy acted as a moderator for the effectiveness of NRT in their trial. Therefore, including cognitive behavioral intervention targeting self-efficacy such as the interactive web-based intervention which targets the self-efficacy in addition to NRT, can lead to various degrees of success for treating tobacco use and nicotine dependence^30^. While the social support provided through coach calls or web-based program in combination with NRT was shown in two interventions with large sample size and a small effect^17,24^. A similar finding was supported in a previous review^31^.

The dose of NRT is another aspect which could increase the likelihood of OST abstinence. This was shown in two included interventions, one of them had a small effect with statistically significant difference from a large sample size^17^. While the other one was supporting this finding although it failed in reporting sufficient data for the effect calculation^21^. These two studies found that the high dose of NRT was associated with increased OST abstinence rate as a result of a greater reduction in tobacco withdrawal symptoms in comparison to the low dose. The used measure of outcome assessment can influence the interpreted effect of NRT by overestimating of the OST abstinence and reduction rate, which could be the case when self-report was used as outcome measurement.

Sample size could be another contributor to the efficacy of the intervention. For example, two of the included interventions had a large sample size and reported a small NRT effect^17,24^. While two interventions with small sample size had a moderate NRT effect^23,25^. Home setting and web-based setting were associated with large sample size. While one of the interventions done in a healthcare setting and another intervention at a workplace setting were associated with small sample size. When comparing the effect sizes among the settings, the medium effect size of NRT was found in the healthcare setting and workplace setting, although the latter did not show statistical significance. In contrast, interventions delivered in home or web-based settings had a smaller effect size when compared to the other settings. However, the role of setting in most of the included interventions was not clearly emphasized; the intervention was targeting only individuals by providing NRT alone or in combination with elements through coach calls or web-based interactive page for social support and self-efficacy. Contextual elements such as having a partner who uses OST at home – which could negatively influence participant outcomes – were not taken into consideration. This could explain the small effect of NRT found at home setting when compared to the intervention at workplace setting which had a moderate effect size of NRT on OST abstinence.

### Limitations

There are few limitations in this review. Some were related to the review and the others were related to the included studies. The review included only studies in the English language. This may result in the exclusion of relevant interventions published in other than English. Whereas most of the included studies were varied in quality from low to high, most studies had small sample size and only two had large sample size, participants were predominantly male, used different comparison groups, done at different settings, most of the studies were done in the USA, and some of the studies did not report the related findings of NRT effect. It was difficult to focus on one setting than the others due to the limited number of studies on this subject.

### Future research

If these interventions would be carried out in the future, researchers and healthcare providers should incorporate the setting context with NRT to increase the likelihood of OST abstinence rate. The web-based setting has the advantage of reaching a large number of participants than other types of setting. Home setting interventions, where the researchers mail the NRT to the home address of the participants, could also be a good option to reach large sample size. However, one must address the other features in the home setting that could influence the success of NRT in OST abstinence and reduction. Such features could be related to the home environment, the presence of a partner using tobacco or the negative social pressure. Community-based interventions can lead to a significant impact if planned carefully. Further future investigations including larger sample size with more emphasis on setting context are required to assess NRT effect on OST abstinence and reduction rate.

## CONCLUSIONS

This systematic review contributed to the understanding of the effectiveness of NRT on OST cessation and reduction. Overall, NRT can aid OST users in quitting. Combination of NRT with other interventions such as coach calls and web-based interactive setting can enhance the OST abstinence rate^31^. Moreover, this review pointed out some aspects such as nicotine dependence level, NRT dose, self-efficacy, social support and context of the setting that could have contributed to the effectiveness of NRT interventions.

## Supplementary Material



## Data Availability

The data supporting this research can be found in the Supplementary file.
